# Pulmonary vein reconnection rates after pulse field ablation: time for a reality check?

**DOI:** 10.1093/europace/euaf014

**Published:** 2025-01-17

**Authors:** Alessio Gasperetti, Luigi Di Biase

**Affiliations:** Department of Medicine, Division of Cardiology, Johns Hopkins University, Baltimore, MD, USA; Cardiac Arrhythmia Center, Division of Cardiology at Montefiore-Health System, Albert Einstein College of Medicine, 111 East 210th Street, Bronx, New York, NY 10467, USA

**This editorial refers to ‘Repeat Procedures After Pulsed Field Ablation for Atrial Fibrillation: *MANIFEST-REDO* Study’, by D. Scherr *et al*., https://doi.org/10.1093/europace/euaf012.**

Since the turn of the millennium, the electrical contribution of the pulmonary veins (PVs) has been recognized as critical for the generation and sustenance of atrial fibrillation (AF).^1^ Consequentially, pulmonary vein isolation (PVI) has become the cornerstone of current ablation strategies for AF, on the top of which all additional ablative strategies have been devised. Whether an operator is a believer,^2^ a worshipper of VENUS,^3^ or a sub-CAPLA follower,^4^ the common procedural step always agreed upon is represented by the performance of PVI as a starting point.

On the method of PVI performance, much has been written. Radiofrequency and cryo-energy have for years represented the most commonly used modalities, but in the past few years, the field of AF ablation has been overrun by the excitement for the new kid on the block: pulse field ablation (PFA). This new form of ablation energy is based on the use of selective, myocardial-specific electroporation and harbours the promise of shorter procedure times, a better safety profile, and comparable (if not improved) clinical outcomes.^5–7^ While randomized and non-randomized clinical and outcome-based studies have been flourishing,^8,9–12^ reports addressing lesion durability and rates of PVI isolation at long-term follow-up with PFA-based technologies outside of clinical trials (in the so called ‘real-world’ setting) have been scarce.

In this issue of *Europace*, Scherr and colleagues^13^ come to the rescue and shed additional light on this topic, addressing the real-world success rate of PVI using a pentaspline PFA catheter by leveraging the data from the prospective, international, patient-level MANIFEST PF registry.^13^ All investigators should be congratulated for their effort. Their study presents the procedural and long-term data of the 427 patients (64 ± 11 years of age, 63% male) enrolled in the registry that underwent a first re-map and re-do AF ablation procedure due to an atrial arrhythmia recurrence (51% pAF, 30% pxAF, and 19% AT), after undergoing PVI with a PFA pentaspline catheter at the time of index procedure. Their results are of great interest: at 281 ± 176 days after the initial PFA procedure, only 44% of patients with a clinical recurrence had all PV durably isolated, while 29%, 16%, 9%, and 1% had, respectively, 1, 2, 3, or all PVs reconnected. The repeat procedure yielded a good clinical impact, with freedom from atrial arrhythmias being achieved in 64.6% of patients after re-do procedure at a median of 284 days of follow-up. Posterior wall isolation showed only a trend towards improved outcome in patients with pxAF (68.3% vs. 51.2%, *P* = 0.07), while pxAF was the strongest multivariate predictor of recurrence at long-term follow-up.

The main message from the manuscript is two-fold. First, the PVI lesion durability with PFA in the real world with current technologies is lower than originally expected. Initially, PFA premarket studies with per-protocol mandatory remapping had shown a 96% durability of isolation at a vein level and an 84% durability of complete isolation at patient level.^10^ These data had led to an hyperinflated enthusiasm for PFA, as they came with the promise that PFA could create durable, transmural lesion in the vast majority of patients, hence *improving* outcomes of AF ablation. Instead, we here have now the second large sample size report published that shows relatively sub-optimal PVI rates in the real-world setting,^11^ with overall vein reconnection levels at repeat procedures in line with the ones seen with other forms of energy.^12^ PFA has unquestionable multiple comparative advantages (safety and learning curve above all), but these data seem to warrant a reshape of the narrative that we are spinning for this new technology. It is fair to say that the presence of intracardiac echo and 3D mapping aiming to address contact could represent a factor able to improve outcomes and PV reconnection. Nontheless, jury seems to be still out on wether PFA is actually improving patients' outcome. Secondly, these data further strongly support a vein-centred paradigm of AF ablation. Regardless of the energy employed, across the board, PV reconnection seems by far to represent the main driver of AF recurrence. No re-do procedure would be complete without a testing and re-isolation of the reconnected veins, as they represent the main procedural target. However, while the paradigm of AF ablation is (and will still be for the foreseeable future) PV centred, it is not PV exclusive. This manuscript reminds us that our fight with AF is not over: almost half of patients with a recurrence did not have any PV reconnection, reminding us of the existence of non-PV triggers and targets. In this, PFA still harbours the promise of a potential quantum leap in the field, due to its allowance of more aggressive (as more myocardial specific) extra PV ablation protocols.

In conclusion, we congratulate Dr Scherr^13^ and colleagues for their great contribution to the field of AF ablation, as they remind us of the central role of PV in AF pathogenesis and for their reality check on the real-world effectiveness of PFA.

## Data Availability

No data was used for this manuscript.
